# The predominant role of FliC contributes to the flagella-related pathogenicity of ST34 *S.* Typhimurium monophasic variant

**DOI:** 10.1186/s13567-024-01427-2

**Published:** 2024-12-18

**Authors:** Yaming Hong, Qilong Hou, Hui Liu, Xiaojie Wang, Jiaojie Gu, Zhenyu Wang, Xinan Jiao, Qiuchun Li

**Affiliations:** 1 Jiangsu Key Lab of Zoonosis/Jiangsu Co-Innovation Center for Prevention and Control of Important Animal Infectious Diseases and Zoonoses, Yangzhou University, Yangzhou, China; 2 Key Laboratory of Prevention and Control of Biological Hazard Factors (Animal Origin) for Agri-Food Safety and Quality, Ministry of Agriculture of China, Yangzhou University, Yangzhou, China; 3 Joint International Research Laboratory of Agriculture and Agri-Product Safety, Yangzhou University, Yangzhou, China

**Keywords:** *Salmonella enterica* serovar Typhimurium (*S*. Typhimurium), *S*. Typhimurium monophasic variant, ST34, FliC, FljB

## Abstract

Over the past two decades, the monophasic variant of *Salmonella enterica* serovar Typhimurium (*S*. Typhimurium) has rapidly emerged and increased worldwide. This upsurge is especially true for the European clone of the ST34 *S*. Typhimurium monophasic variant. The key distinction between ST34 *S*. Typhimurium and its monophasic variant is that the genes that encode for second-phase flagellin (FljB) and the regions around it have been replaced with various multidrug resistance cassettes. To determine if the loss of *fljB* or the retention of *fliC*,-the gene coding for first-phase flagellin (FliC)-, would impact its pathogenicity, we constructed various mutations, including deletions of *fljB*, *fliC*, *fliC*/*fljB*, and strains where *fliC* was replaced with *fljB*. Our results showed that the loss of *fljB* in ST34 *S*. Typhimurium and its monophasic variant does not affect bacterial motility, cell infection ability, survival in macrophages, induced pro-inflammatory cytokines secretion, virulence, or persistent infection in mice. However, the deletion of *fliC* caused a significant decrease in these outcomes for both strains, while the replacement of *fliC* with *fljB* only partially restored these capabilities. Consequently, we determined that FliC is predominant in the flagellar expression of ST34 *S*. Typhimurium other than FljB. This finding demonstrates that replacing the *fljB* gene with various resistance regions in ST34 *S.* Typhimurium monophasic variants can enhance bacterial survival under specific antibiotic farming practices and spread globally.

**Supplementary Information:**

The online version contains supplementary material available at 10.1186/s13567-024-01427-2.

## Introduction

*Salmonella enterica* is one of the most prevalent pathogens responsible for causing human enteric diseases globally [1]. To date, over 2600 serovars have been identified and reported to be closely associated with various animals [2]. Among these serovars, *Salmonella enterica* serovar Typhimurium (*S*. Typhimurium) is one of the most commonly reported serovars responsible for human salmonellosis cases and outbreaks worldwide [3]. However, over the past two decades, a new *Salmonella* serotype, the *S*. Typhimurium monophasic variant (*Salmonella* 4,[5],12:i:-), has rapidly emerged and become one of the most common serotypes responsible for multiple foodborne outbreaks [4]. Although three clones have been identified in monophasic *S*. Typhimurium, the ST34 European clone, especially, has spread and caused worldwide infections in pigs and humans [5]. Notably, multiple outbreaks of foodborne diseases caused by monophasic *S*. Typhimurium have been reported in Luxembourg, Italy, and France [6–8]. In 2022, chocolate contaminated with the ST34 *S*. Typhimurium monophasic variant caused global infections, with 89% of cases involving children [9, 10].

*S*. Typhimurium is biphasic and can produce either the first-phase flagellin (FliC) or the second-phase flagellin (FljB) through phase switching, controlled by the H segment [11]. However, the ST34 European clone *S*. Typhimurium monophasic variant cannot produce second-phase flagellar antigen due to the replacement of the *fljAB* operon and its neighbouring regions by various resistance regions through IS*26*/IS*1R*-mediated transpositions [12, 13]. Flagellin is a structural component of bacterial flagella that facilitates bacterial swimming and enables the directed movement of bacteria toward nutrients or infection sites [14].

Flagella are recognised as pathogen-associated molecular patterns (PAMP) that bind to the toll-like receptor 5 (TLR-5) to activate the NF-κB pathway, resulting in the production of cytokines and chemokines [15]. The flagellin structures of FliC and FljB have been found to have four interconnected domains: D0, D1, D2, and D3 [14]. The major differences between the two flagellins are located in the central D2 and D3 domains [16, 17]. FliC-expressed *S.* Typhimurium has shown distinct motility on the cell host surface compared to FljB-expressed *S.* Typhimurium and has a competitive advantage in colonisation within intestinal epithelia [10].

Nonetheless, it remains unclear whether the loss of *fljB* in the *S*. Typhimurium monophasic variant affects bacterial motility, virulence, and inflammatory responses. Understanding this may explain its prevalence in animals and humans, particularly when different resistance regions replace *fljB*.

Our previous study explained the molecular mechanism underlying the emergence of the European clones of the *S.* Typhimurium monophasic variant from ST34 *S*. Typhimurium [18]. Both in vitro and in vivo experiments confirmed that the *S.* Typhimurium YZU0463 strain can be induced into the *S*. Typhimurium monophasic variant YZU2855 strain through IS*26*-mediated transpositions [18].

In this study, we further constructed *fliC*-deleted mutants, YZU0463Δ*fliC* and YZU2855Δ*fliC*, and replaced *fliC* with *fljB* in the native chromosomal locus of YZU2855 strain (YZU2855 ^*fliC*→*fljB*^). Furthermore, we used the control ST19 *S*. Typhimurium strain SL1344 to construct mutants with the deletion of *fliC*, *fljB*, and both genes. The bacterial motility, cell infection ability, survival within macrophages, induction of pro-inflammatory cytokines secretion, cytotoxicity, and virulence in mice were examined to demonstrate the role of *fliC* and *fljB* in ST34 *S.* Typhimurium and its monophasic variant.

## Materials and methods

### Bacterial strains and growth condition

The ST19 *S*. Typhimurium strain SL1344 was acquired from the National Institute for the Control of Pharmaceutical and Biological Products (Beijing, China). The ST34 *S*. Typhimurium strain YZU0463 and its induced *Salmonella* 4,[5],12:i:- strain YZU2855 were obtained as previously described [18]. All bacterial strains and plasmids utilised in this study are listed in Additional file 1. Deletion of *fliC* or *fljB* in *Salmonella* strains was constructed by a double exchange of homologous recombination using the pDM4 plasmid, as previously described [19]. All primers used to construct recombinant bacteria are listed in Additional file 2. Bacteria were cultured on Luria–Bertani (LB) agar or in LB broth at 37 ℃ with agitation at 180 rpm.

### Bacterial motility assays

Swimming assays were performed on semisolid LB plates with 0.3% w/v agar. Overnight cultures were grown in liquid LB medium at 37 ℃ with shaking at 180 rpm. Bacteria cultures were collected and washed twice with sterile phosphate-buffered saline (PBS), then normalised to an OD_600_ of 1.0 for inoculation on LB agar plates. A 10 μL aliquot of the normalised bacterial culture was inoculated onto semisolid LB agar plates and incubated at 37 ℃. The widest diameter of each colony was measured after 10 h of incubation. The swimming plates were visualised using a Bio-Rad GelDoc 2000 imaging system (Bio-Rad, USA). Each assay was performed thrice. All *S*. Typhimurium and *Salmonella* 4,[5],12:i:- strains listed in Additional file 1 were involved in the bacterial motility assays.

### Gene expression analyses

Bacterial overnight cultures were diluted at 1:100 into LB broth and grown at 37 ℃ with shaking at 180 rpm until they reached the logarithmic phase (OD_600_ = 0.7). The bacterial cultures were then subjected to total RNA extraction using an RNAprep Pure Bacteria Kit (Tiangen, Beijing, China) according to the manufacturer’s instructions. The RNA was reverse transcribed to cDNA using HiScript III RT SuperMix (Vazyme, Nanjing, China) and then subjected to real-time qPCR. The real-time qPCR was performed using the QuantStudio 6 Flex Real-Time System (Applied Biosystems, Foster City, USA) with primers listed in Additional file 2.

The qRT-PCR reaction was performed in a total volume of 20 μL, containing 10 μL of 2 × AceQ Universal SYBR qPCR Master Mix (Vazyme), 400 ng of cDNA, 0.2 μL each of 10 μM forward and reverse primers, and 7.2 μL RNase-free water (Vazyme). The comparative threshold (2^–ΔΔC(T)^) method was used to calculate the relative expression levels of these genes. All the real-time qPCR assays were performed thrice. The expression of *fliC* was tested in YZU0463, YZU2855, SL1344, and SL1344Δ*fljB*. The expression of *fljB* was tested in YZU0463, YZU0463Δ*fliC*, YZU2855^*fliC*→*fljB*^, SL1344, SL1344Δ*fliC*, and SL1344Δ*fljB *^*fliC*→*fljB*^.

### Cell infection assays

The HeLa cells and RAW264.7 macrophages were cultured at 37 ℃ with 5% CO_2_ in Dulbecco’s modified Eagle medium (DMEM) (Gibco, Grand Island, USA) supplemented with 10% foetal bovine serum (FBS) (Gibco), 100 μg/mL penicillin, and streptomycin (Gibco) [20]. IPEC-J2 cells were cultured in RPMI 1640 medium (Gibco) containing 10% FBS, penicillin, and streptomycin (100 μg/mL) [21]. All the cells were obtained from the Jiangsu Key Lab of Zoonosis (Yangzhou University, China). Trypan blue exclusion assays measured cell viability and number.

Cells were seeded at 2.5 × 10^5^ per well into 24-well plates and cultured for approximately 12 h to reach 80% confluence. Overnight bacterial cultures were normalised to an OD_600_ of 1.0 and used to infect HeLa and IPEC-J2 cells at a multiplicity of infection (MOI) of 100 and RAW264.7 macrophages at an MOI of 10. The plates were centrifugated at 1000 rpm for 10 min to promote bacterial interaction with cells. After 30 min of incubation, the cells were lysed using 0.1% Triton X-100 and serial 1:10 dilutions of the lysate were plated on LB agar to calculate the number of adhesive bacteria. The adhesion rate was determined by calculating the ratio between the number of adhesive bacteria and the number of bacteria initially infected.

During the cell invasion assays, after 30 min of incubation, the medium containing 100 μg/mL gentamicin was used to kill extracellular bacteria. A further 1 h of incubation then followed. The cells were then lysed, and serial 1:10 dilutions of the lysate were plated on LB agar to calculate the number of invasive bacteria. The invasion rate was determined by calculating the ratio between the number of invasive bacteria and the number of bacteria initially infected.

After 1 h of incubation in a medium containing 100 μg/mL gentamicin, the bacterial proliferation assay had the medium replaced with one containing 10 μg/mL gentamicin for an additional 6 h of incubation. The cells were then lysed, and serial 1:10 dilutions of the lysate were plated on LB agar to calculate the number of intracellular bacteria. Each strain was used to infect three wells of cells, and the experiment was performed thrice. All *S*. Typhimurium and *Salmonella* 4,[5],12:i:- strains listed in Additional file 1 were involved in the cell infection assays.

### Detection of pro-inflammatory cytokines and lactate dehydrogenase (LDH) release in infected cells

The HeLa and RAW264.7 cells were used to detect cytotoxicity and the release of cytokines following bacterial infection. At 3 and 6 h post-infection, the cultures’ supernatants were collected for the detection of LDH and pro-inflammatory cytokines. LDH was quantified using the LDH Cytotoxicity Assay Kit (Beyotime Biotechnology Co. Ltd, China) according to the manufacturer’s instructions. The release of pro-inflammatory cytokines was analysed using the CBA human/mouse inflammatory cytokines kit (BD, USA) as per the manufacturer’s instructions. Cytokine expression levels were detected using the BD FACSAria SORP (BD, USA). Each assay was performed thrice.

### Mouse experiments

Female BALB/c mice (6–8 weeks old) were obtained from the Institute of Comparative Medicine at Yangzhou University and bred under specific pathogen-free (SPF) conditions. The Yangzhou University Animal Welfare and Ethics Committees approved all animal experiments conducted in this study (NSFC2019-SJXY-4).

For the bacterial colonisation assay, 144 BALB/c mice were randomly divided into six groups, each with 24 mice. Five groups of mice were infected with YZU0463, YZU2855, YZU0463Δ*fliC*, YZU2855Δ*fliC*, and YZU2855^*fliC*→*fljB*^, respectively. A negative group was treated with 100 μL PBS by oral gavage. Before infection, mice were pretreated with one 7.5 mg dose of streptomycin to minimise the impact of gut commensal bacteria. Food and water were withheld for 5 h before oral gavage.

The experimental group was orally infected with 100 μL of bacteria diluted in PBS at 1 × 10^7^ CFU per mouse. At 3, 7, and 14 days post-infection (dpi), the tissues of five mice were harvested and homogenised. The bacterial load in the liver, spleen, ileum, cecum, and Peyer’s patches was determined by plating on brilliant green agar (Thermo, USA) after tenfold serial dilution of the tissue homogenate. At 14 dpi, the livers were harvested, fixed in 4% neutral buffered paraformaldehyde for 24 h, and then embedded in paraffin. The sections were stained with haematoxylin–eosin (H&E). Images were captured using a panoramic slice scanner (3DHISTECH, Hungary).

To assess the virulence of the YZU0463, YZU2855, YZU0463Δ*fliC*, YZU2855Δ*fliC,* and YZU2855^*fliC*→*fljB*^ strains, a total of 30 mice were divided into six groups (*n* = 5) and orally infected with 1 × 10^8^ CFU of bacteria. The survival of each group of mice was monitored for 15 days.

### Statistical analysis

All data were presented as mean ± SEM of triplicate samples from three independent experiments using GraphPad Prism 6.0 software. A two-way ANOVA with Tukey’s test was performed to determine the statistical significance, with *P* values < 0.05 considered significant.

## Results

### The *fliC*-encoded type I flagellar is crucial for swimming motility

**Figures 1:**
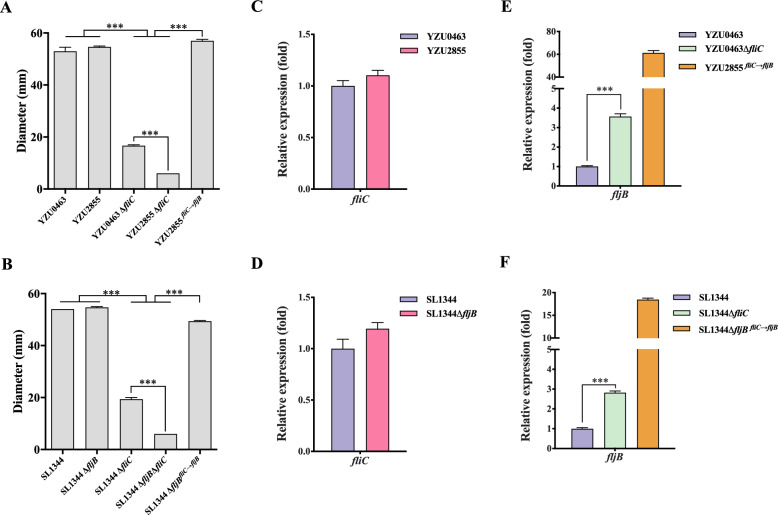
**The locus of** ***fliC* is required for bacterial swimming motility**. **A** Diameters of cell spread were measured 10 h post-inoculation of *S*. Typhimurium ST34 strain YZU0463 and its various mutants on swimming plates. **B** Diameters of cell spread were measured 10 h post-inoculation of *S*. Typhimurium ST19 strain SL1344 and its derived mutants on swimming plates. **C, E** qRT-PCR analysis of *fliC* in YZU0463, YZU2855 and *fljB* in YZU0463, YZU0463Δ*fliC,* YZU2855^*fliC*→*fljB*^. **D, F** qRT-PCR analysis of *fliC* in SL1344, SL1344Δ*fljB* and *fljB* in SL1344, SL1344Δ*fliC,* SL1344Δ*fljB*^*fliC*→*fljB*^*.* Each assay was performed in triplicate. Error bars represent ± SEM. ***P < 0.001.

The swimming assay demonstrated that the swimming zone diameters of ST34 *S*. Typhimurium YZU0463 were comparable to those of its monophasic variant YZU2855 (Figure 1A, Additional file 3) and, similarly, to those of the ST19 *S*. Typhimurium SL1344 and its *fljB*-deleted strain SL1344Δ*fljB* (Figure 1B, Additional file 3). The deletion of *fliC* in *S*. Typhimurium YZU0463 and SL1344 resulted in a significant decrease in the swimming zone diameters by over twofold when compared to the wild-type (WT) strains (Figure 1A and B, Additional file 3). Furthermore, the deletion of *fliC* in the monophasic variants YZU2855 and SL1344Δ*fljB* caused approximately a fourfold reduction in the swimming zone diameters (Figures 1A and B, Additional file 3). However, replacing *fliC* with *fljB* in YZU2855 and SL1344Δ*fljB* strains restored their swimming ability to the same level as that of the YZU0463 and SL1344 strains (Figures 1A and B, Additional file 3).

Subsequently, the expression of *fliC* in YZU0463 and YZU2855 was detected using qRT-PCR. The results indicated that the expression levels of *fliC* are similar in both strains, which is consistent with observations in the SL1344 and SL1344Δ*fljB* strains (Figures 1C and D). However, qRT-PCR analysis showed that *fljB* expression in the *fliC*-deleted YZU0463Δ*fliC* and SL1344Δ*fliC* strains had an approximately threefold expression increase compared to the WT strains (Figures 1E and F). In comparison, *fljB* expression showed approximately more than 60-fold and 15-fold increases in the YZU2855^*fliC→fljB*^ and SL1344Δ*fljB*^*fliC→fljB*^ strains, respectively, after replacing *fliC* with *fljB*, (Figures 1E and F).

These results indicate that *fliC* is highly expressed to encode type I flagellar in both *S*. Typhimurium and its monophasic variant. This finding is closely related to bacterial swimming ability (Additional file 3).

### Deletion of *fliC* impairs bacterial adhesion and invasion of host cells

**Figure 2:**
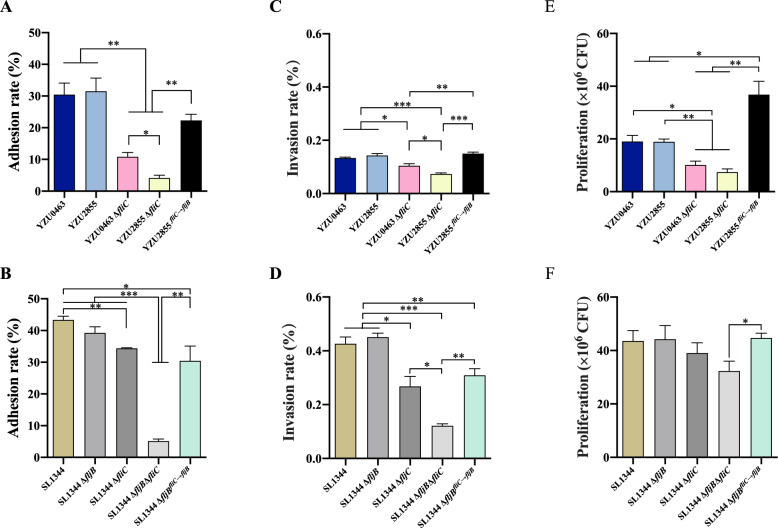
**Deletion of *fliC* impairs bacterial adhesion and invasion in HeLa cells**. **A, C, E** HeLa cells were infected with ST34 *S*. Typhimurium and *Salmonella* 4,[5],12:i:- strains. **B, D, F** HeLa cells were infected with ST19 *S*. Typhimurium and its derived mutants. Each assay was performed in triplicate. Error bars represent ± SEM. *P < 0.05; **P < 0.01; ***P < 0.001.

To evaluate how the expression of *fliC* or *fljB* affects the bacterial invasion of epithelial cells, we infected HeLa cells with *S.* Typhimurium and its monophasic variant to quantify the levels of adherent and intracellular bacteria. We observed that the monophasic YZU2855 strain, which lacks *fljB*, exhibited a similar adhesion rate to the parental YZU0463 strain (Figure 2A). However, the adhesion rate of the *fliC-*deleted YZU0463Δ*fliC* strain was approximately 10% compared to 30% for the YZU0463 strain (Figure 2A).

The *fliC*-deleted YZU2855Δ*fliC* strain adhesion rate was approximately 4% compared to 10% for the YZU0463Δ*fliC* strain (Figure 2A). Replacement of *fliC* with *fljB* in YZU2855 partially restored its adhesion ability (Figure 2A). However, in the ST19 *S*. Typhimurium SL1344 strain, the deletion of *fljB* did not affect the bacterial adhesion to HeLa cells (Figure 2B). In comparison, the deletion of *fliC* caused a slight reduction in the ability to adhere to cells (Figure 2B). Notably, the deletion of both *fliC* and *fljB* dramatically reduced adhesion to cells (Figure 2B).

Bacterial invasion assays revealed that the Δ*fljB* mutants exhibited an invasion rate comparable to the WT strains (Figures 2C and D). However, both the Δ*fliC* and Δ*fljB*Δ*fliC* mutants showed significantly decreased invasion rates. Furthermore, the Δ*fljB*Δ*fliC* mutants displayed an even lower invasion rate than the Δ*fliC* mutants (Figures 2C and D). Replacement of *fliC* with *fljB* in the YZU2855^*fliC*→*fljB*^ and SL1344Δ*fljB*^*fliC*→*fljB*^ strains restored the invasion rate to a level similar to that of YZU0463 and SL1344 (Figures 2C and D). The bacterial invasion results were consistent with the bacterial motility results (Figures 1A and B).

To determine if the bacterial infection ability was specific to human epithelial cells, we utilised the swine intestinal epithelial cell line, IPEC-J2, as the model. Similar to the observed adhesion and invasion rate of HeLa cells, the Δ*fliC* and Δ*fljB*Δ*fliC* mutants displayed decreased adhesion and invasion rates to IPEC-J2 cells compared with the respective WT strains (Additional file 4). Subsequently, we quantified the number of bacteria proliferating in HeLa cells at 6 h post-infection. The results indicated that YZU0463Δ*fliC* and YZU2855Δ*fliC* numbers were significantly lower than YZU0463 and YZU2855 (Figure 2E). A decrease in proliferation was also detected in the SL1344Δ*fljB*Δ*fliC* strain compared to other strains derived from SL1344 (Figure 2F).

### Absence of *fliC* decreases pro-inflammatory immune responses in infected HeLa cells

**Figure 3:**
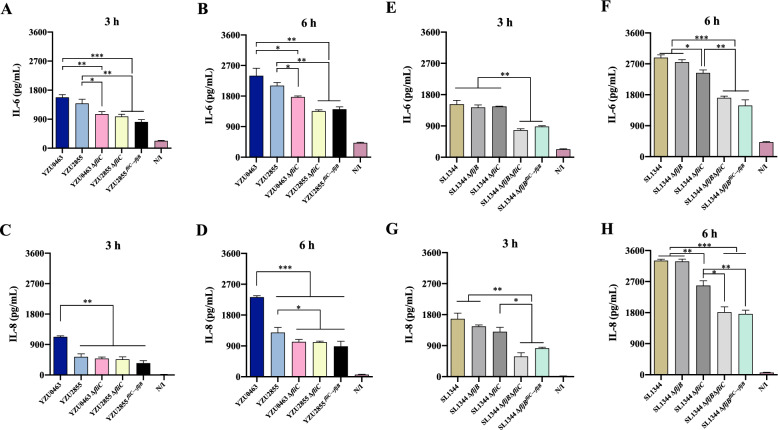
**Deletion of *fliC* reduces IL-6 IL-8 secretion in infected epithelial cells**. The supernatants of cell cultures were harvested 3 h and 6 h post-infection of *Salmonella* for the detection of pro-inflammatory cytokines. **A–D** HeLa cells were infected with ST34 *S*. Typhimurium and *Salmonella* 4,[5],12:i:- strains. **E–H** HeLa cells were infected with ST19 *S*. Typhimurium and *Salmonella* 4,[5],12:i:- strains. The N/I group represents the uninfected group. The experiment was performed three times in duplicates. Error bars represent ± SEM. *P < 0.05; **P < 0.01; ***P < 0.001.

Flagellin of *Salmonella* can activate the innate immune responses via specific interactions with Toll-like Receptors (TLRs), thus producing pro-inflammatory cytokines. We measured the expression levels of IL-6 and IL-8 in the supernatant of HeLa cells infected with different *Salmonella* strains. The secreted IL-6 levels in both YZU0463- and YZU2855- infected cells were similar and significantly higher than those in cells infected with YZU0463Δ*fliC*, YZU2855Δ*fliC*, and YZU2855^*fliC*→*fljB*^ at both 3 h and 6 h post-infection (Figures 3A and B). These findings suggest that FliC plays a significant role in promoting IL-6 secretion in HeLa cells during infection. Nonetheless, IL-8 secretion was significantly lower in HeLa cells infected with the YZU2855 or *fliC*-deleted strains than those infected with the *fliC*-positive YZU0463 strain. Replacing *fliC* with *fljB* did not recover IL-8 secretion (Figures 3C and D). Interestingly, in cells infected with the ST19 *S*. Typhimurium strain, deletion of *fljB* did not affect IL-6 and IL-8 secretion (Figures 3E–H). The deletion of *fliC* led to decreased IL-6 secretion at 6 h post-infection but not at 3 h post-infection (Figures 3E and F). However, the double mutant SL1344Δ*fljB*Δ*fliC* and the *fljB*-replaced strain SL1344Δ*fljB*^*fliC*→*fljB*^ showed dramatically decreased secretion of both IL-6 and IL-8 (Figures 3E–H), indicating *fljB* cannot replace *fliC* in inducing inflammatory responses.

### Deletion of *fliC* facilitates bacterial replication in infected macrophages due to decreased inflammatory responses

**Figure 4:**
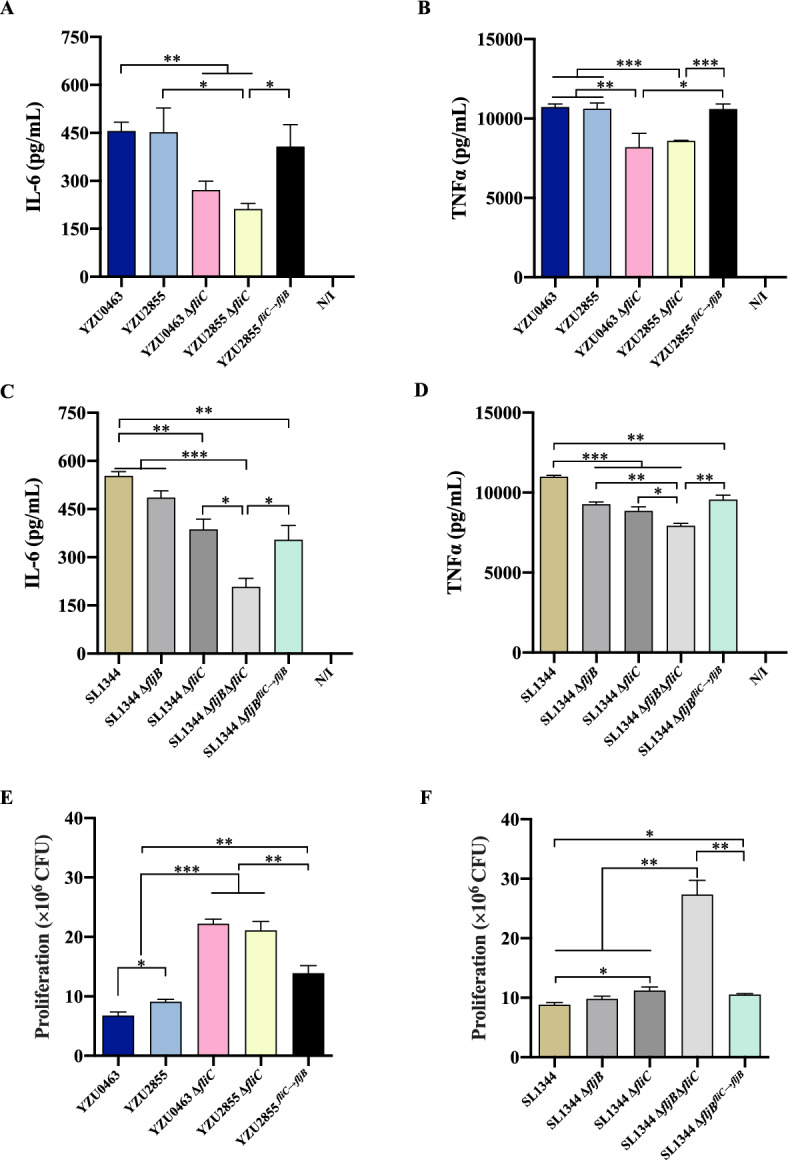
**Deletion of *fliC* facilitates bacterial replication and reduces IL-6 and TNF-α secretion in macrophages**. **A, B, E** RAW264.7 cells were infected with ST34 *S*. Typhimurium and *Salmonella* 4,[5],12:i:- strains. **C, D, F** RAW264.7 cells were infected with ST19 *S*. Typhimurium and *Salmonella* 4,[5],12:i:- strains. The N/I group represents the uninfected group. The experiment was performed three times in duplicates. Error bars represent ± SEM. *P < 0.05; **P < 0.01; ***P < 0.001.

It is widely recognised that *S.* Typhimurium can infect macrophages and induce the production of pro-inflammatory cytokines [22]. In this study, flow cytometry measured the cytokine secretion levels of macrophage RAW264.7 cells infected with different *Salmonella* strains at 6 h post-infection. Consistent with the results of IL-6 levels secreted by HeLa cells infected with ST34 *Salmonella* strains, the loss of *fljB* in the monophasic variant YZU2855 did not affect IL-6 and TNFα levels compared to the *S*. Typhimurium strain YZU0463. Conversely, the deletion of *fliC* in both strains caused a significant decrease in IL-6 and TNFα secretion compared to cells infected with WT strains. Replacing *fliC* with *fljB* in YZU2855 can restore the secretion of IL-6 and TNFα in infected macrophages, but this response was not observed in infected HeLa cells (Figs. 3A and B, 4A and B).

In ST19 *S*. Typhimurium infected macrophages, the deletion of *fliC* in SL1344 significantly decreased the secretion of IL-6 and TNFα. Further deletion of *fljB* in the SL1344Δ*fliC* strain caused an even greater reduction in the secretion of both IL-6 and TNFα. Replacing *fliC* with *fljB* in the SL1344Δ*fljB* mutant only restored the secretion levels of IL-6 and TNFα to those observed in SL1344Δ*fliC*-infected macrophages (Figures 4C and D). Subsequently, we measured the bacterial count in infected macrophages at 6 h post-infection. The *fliC*-deleted mutants of ST34 *S.* Typhimurium strains (YZU0463Δ*fliC* and YZU2855Δ*fliC*) exhibited over a twofold increase in bacterial numbers compared to the WT strains. Similarly, the double mutant Δ*fljB*Δ*fliC* of ST19 *Salmonella* (SL1344Δ*fljB*Δ*fliC*) also showed more than a twofold increase in bacterial numbers compared to the other strains (Figures 4E and F).

### Deletion of *fliC* promotes cell death in RAW264.7 macrophages infected with ST34 *S*. Typhimurium strains

**Figure 5:**
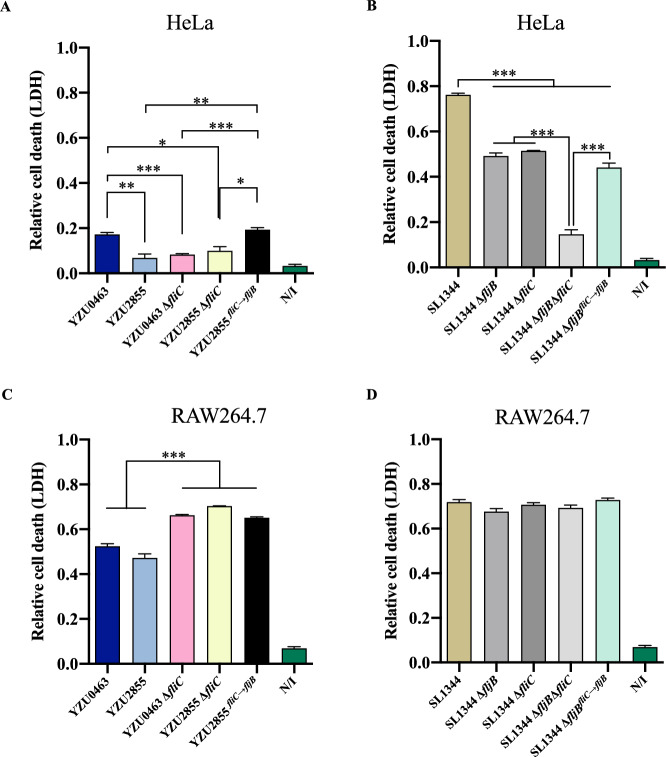
**The release of LDH induced by *fliC* mutants presents opposed changes in HeLa and RAW264.7**. **A, C** Cells were infected with ST34 *S*. Typhimurium and *Salmonella* 4,[5],12:i:- strains. **B, D** Cells were infected with ST19 *S*. Typhimurium and *Salmonella* 4,[5],12:i:- strains. The N/I group represents the uninfected group. Cell cytotoxicity was quantified using the LDH Cytotoxicity Assay Kit. The experiment was performed three times in duplicates. Error bars represent ± SEM. *P < 0.05; **P < 0.01; ***P < 0.001.

It is widely known that *Salmonella* can interact with numerous cell types and frequently cause cell death [23]. In this study, we investigated the effect of flagellin on the release of lactate dehydrogenase (LDH) in epithelial cells and macrophages infected with different *Salmonella* strains. Our findings indicate that in infected HeLa cells, the ST34 *Salmonella* strains induced lower levels of released LDH than the ST19 *Salmonella* strains despite deleting either *fljB* or *fliC,* resulting in decreased LDH levels in ST34 *Salmonella* strains (Figures 5A and B).

The deletion of *fljB* or *fliC* in the ST19 *Salmonella* strain, SL1344, led to similar decreases in LDH release levels compared to the WT strain. However, the double mutant SL1344Δ*fljB*Δ*fliC* induced a considerably decreased release of LDH compared to the single gene mutants (Figure 5B). In infected macrophage RAW264.7 cells, ST34 and ST19 *Salmonella* strains induced high levels of released LDH (Figures 5C and D). Nonetheless, deleting *fliC*, *fljB*, or both did not affect ST19 *Salmonella* strains (Figure 5D). The deletion of *fliC* in YZU0463 or YZU2855 strains resulted in increased LDH release, while no difference was detected between YZU0463 and YZU2855 (Figure 5C). Additionally, the replacement of *fliC* with *fljB* in the YZU2855 strain did not decrease LDH release, indicating that *fljB* has no effect on LDH release in infected macrophages (Figure 5C).

### *FliC* plays a predominant role in the colonisation and virulence of ST34 *S*. Typhimurium in mice

Previous studies have revealed that the *S.* Typhimurium monophasic variant derived from ST34 *S*. Typhimurium increases the colonisation ability in mice [18, 24]. Therefore, we further compared the effects of FliC and FljB on ST34 *S.* Typhimurium colonisation and virulence in BALB/c mice.

**Figure 6:**
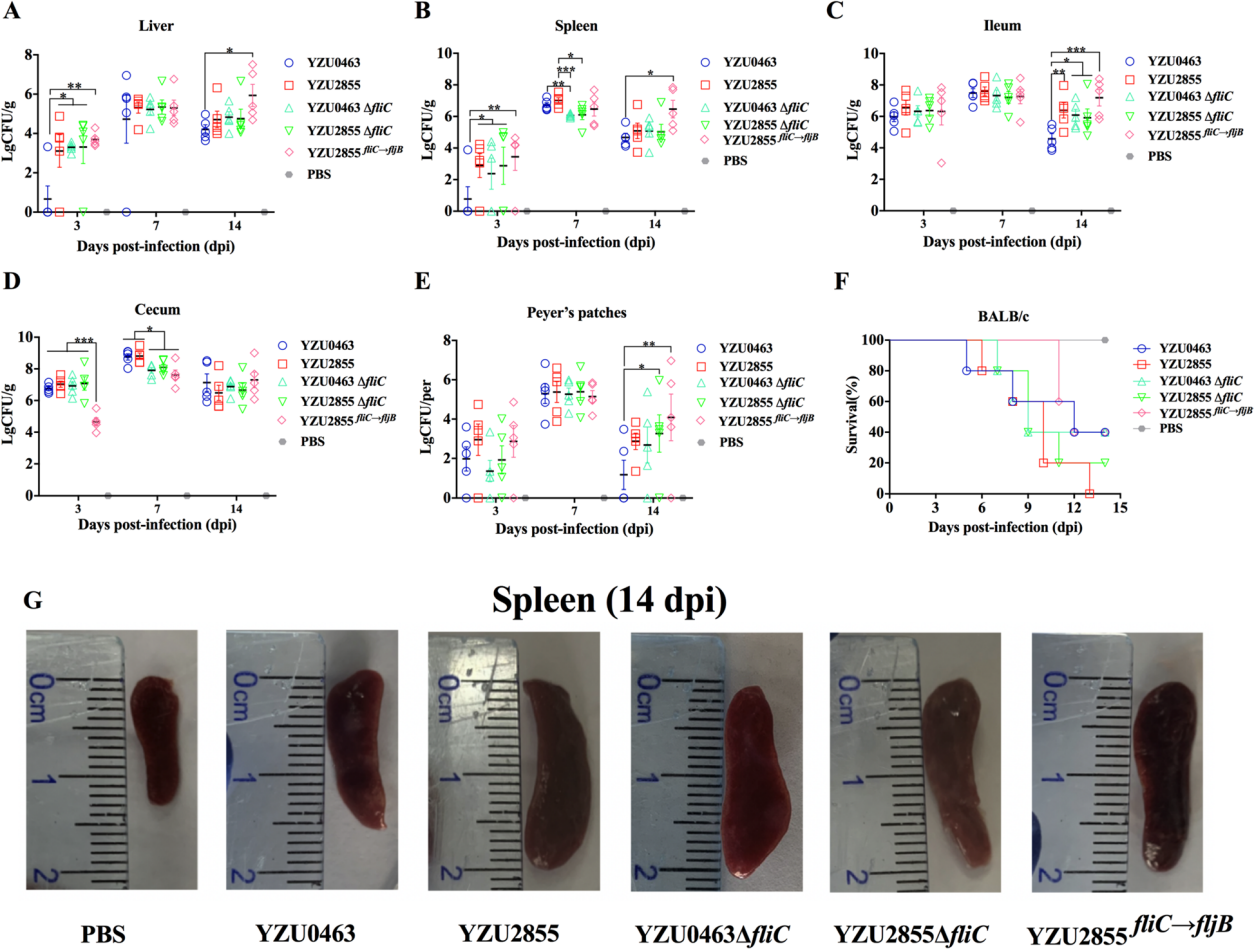
**Deletion of the flagellin gene alters colonisation and virulence in BALB/c mice**. BALB/c mice were infected with ST34 *S*. Typhimurium and *Salmonella* 4,[5],12:i:- strains. At 3, 7, and 14 days post-infection, the bacterial loads per gram of tissue in the liver **(A)**, spleen **(B)**, ileum **(C)**, cecum **(D)**, and Peyer’s patches **(E)** were examined. Each dot represents the count from a single mouse, and the geometric mean is indicated by the horizontal line. Error bars represent ± SEM. *P < 0.05; **P < 0.01; ***P < 0.001. **F** Each of the five BALB/c mice in the six groups was monitored for 15 days. Virulence was determined by the survival rate of mice in each group after infection. **G** Spleen swelling was assessed for comparison among the groups at 14 dpi.

When using 0.1LD50 of YZU0463 strains, the YZU2855 strain, which is deficient of *fljB,* showed increased colonisation in the liver and spleen at 3 dpi (Figures 6A and B) and in the ileum at 14 dpi (Figure 6C). Despite this, the YZU2855 strains caused the death of mice within two weeks when given the dose of 1 × 10^8^ CFU, whereas the YZU0463 strain infected group had a 40% survival rate (Figure 6F). Additionally, the deletion of *fliC* in YZU2855, or replacement of *fliC* with *fljB* in YZU2855, increased the survival rate of mice. In contrast, the deletion of *fliC* in YZU0463 had no significant effect on the survival of mice (Figure 6F). However, bacterial colonisation of YZU2855, YZU0463Δ*fliC*, and YZU2855Δ*fliC* in the liver, spleen, ileum, and Peyer’s patches was higher than in YZU0463, especially in the ileum and Peyer’s patches (Figures 6A–E).

Interestingly, substituting *fliC* with *fljB* enhanced colonisation in the liver, spleen, ileum, and Peyer’s patches at 14 dpi compared to YZU0463 (Figures 6A–E). No significant difference in colonisation was detected among YZU2855, YZU0463Δ*fliC*, and YZU2855Δ*fliC* strains in the liver, spleen, and cecum at 14 dpi (Figures 6A–D). Examination of the spleen sizes from different infected groups at 14 dpi showed that the spleens of mice infected with YZU2855, YZU0463Δ*fliC*, YZU2855Δ*fliC*, and YZU2855^*fliC*→*fljB*^ were larger than those infected with YZU0463. This finding was consistent with the bacterial loads in the spleens (Figure 6G). Notably, haematoxylin and eosin (H&E) staining analysis of the liver at 14 dpi detected liver lesions in mice infected with ST34 *S*. Typhimurium (Additional file 5). Despite the similar levels of tissue damage found in the histological analyses between different infection groups, mice infected with *S*. Typhimurium exhibited extensive steatosis of hepatocytes, lymphocytes and granulocyte infiltration, and significant venous congestion revealing, compared to the uninfected mice (Additional file 5).

## Discussion

In recent years, the ST34 *S.* Typhimurium monophasic variant has become one of the most prevalent serotypes to cause outbreaks of human salmonellosis [25]. Despite this, the factors causing this serotype’s prevalence remain unclear. Furthermore, previous studies have demonstrated that ST34 *S.* Typhimurium cannot be differentiated from its monophasic variant using cgMLST or cgSNPs.

The primary differences between these serotypes are located in the *fljAB* operon and its neighbouring genes [18]. *S*. Typhimurium has two flagellin genes, *fljB* and *fliC*, which are used for flagellar filament formation and to autonomously switch their expression at a frequency of 10^−3^–10^−4^ per cell per generation [26]. Comparably, the *S*. Typhimurium monophasic variant only expresses FliC. Therefore, in this study, we focused on evaluating the effect of FliC and FljB on the biological functions and pathogenicity of ST34 *S.* Typhimurium and its monophasic variant.

Bacterial swimming motility analysis revealed that the ST34 *S*. Typhimurium monophasic variant exhibited comparable motility to ST34 *S*. Typhimurium. Furthermore, qRT-PCR analysis confirmed that the deletion of *fljB* did not affect the expression of *fliC*, suggesting that a loss of *fljB* in the *S*. Typhimurium monophasic variant does not impact bacterial motility in the presence of *fliC*. This outcome aligns with the finding that FliC or FljB expression results in similar bacterial motility [14]. In verification of the bacterial motility results, there was also no difference in the bacterial adhesion, invasion, and survival in epithelial cells between ST34 *S*. Typhimurium and its monophasic variant. However, the loss of *fljB* in the *S*. Typhimurium monophasic variant caused a 50% reduction of IL-8 and LDH secretion in infected HeLa cells compared to ST34 *S*. Typhimurium. Similarly, no significant difference was detected in macrophages infected with the two serotype strains.

Epithelial cells secrete chemokines, such as IL-8, which recruit neutrophils from the circulation into the subepithelial region. This recruitment is a defence against the invasion of *Salmonella* [27]. Therefore, a decrease in the secretion of IL-8 by gut epithelial cells may contribute to the immune escape of the *S*. Typhimurium monophasic variant, leading to increased colonisation in the ileum compared to *S*. Typhimurium in vivo [18]. Furthermore, the *S*. Typhimurium monophasic variant does not elicit significant cellular cytotoxicity, which may facilitate bacterial spread and immune escape [24].

Although deletion of *fliC* in *S*. Typhimurium enhances the expression of *fljB* by approximately threefold, bacterial motility, cell adhesion, and invasion abilities are significantly reduced compared to *fliC*-expressed *S.* Typhimurium. This finding suggests that FliC is predominant in flagellar expression and its related functions. Additionally, the deletion of *fliC* reduced the secretion of pro-inflammatory cytokines but facilitated bacterial replication in infected RAW264.7 cells. These results indicate that the expression of flagellar proteins induces pro-inflammatory cytokines production, which helps to control *S.* Typhimurium proliferation in macrophages. Conversely, the loss of flagellin expression leads to decreased pro-inflammatory cytokine production and increased *S.* Typhimurium proliferation [28]. Previous studies have demonstrated that *Salmonella* expressing FljB exhibited a higher motility than those expressing FliC when subjected to high viscosity conditions [26].

Nevertheless, our results indicated that FljB confers *S*. Typhimurium’s comparable motility to FliC under non-stressful conditions. Replacing *fliC* with *fljB* fully restores the bacterial motility, cell adhesion, and invasion abilities. However, it only partially restores the production of pro-inflammatory cytokines and bacterial proliferation in macrophages. These findings imply that the structural differences between FliC and FljB directly affect their function in inducing immune responses [16, 17]. The central hypervariable region of FliC with the T416A substitution, as found in FljB, significantly reduces recognition by TLR5 and the subsequent expression of pro-inflammatory cytokines [29, 30]. The reduced innate immune response promotes bacterial survival in hosts. Consequently, we found that YZU2855^*fliC*→*fljB*^ exhibited more efficient colonisation in mice than in other groups and had a similar virulence compared to those infected with *fliC*-deleted ST34 *Salmonella* strains.

In conclusion, the increased prevalence of the European clone ST34 *S.* Typhimurium monophasic variant has become a significant global health concern [31]. Our previous studies identified the key difference between ST34 *S.* Typhimurium and its monophasic variant as being located in the region expressing the second-phase flagellar genes. To investigate the impact of this difference, we found that the loss of *fljB* in the ST34 *S.* Typhimurium monophasic variant did not affect *fliC* expression, similar to ST34 *S.* Typhimurium. Additionally, bacterial motility, cell infection ability, survival in macrophages, induced pro-inflammatory cytokines expression, virulence, and persistent infection in mice were comparable between both strains. However, the deletion of *fliC* in both strains caused a dramatic decrease in these abilities compared to the WT strain. Consequently, we have demonstrated that *fliC* expression plays a predominant role in the flagellar-related functions of ST34 *S.* Typhimurium compared to *fljB*. Furthermore, our study has shown that replacing *fljB* with various resistance regions enhances bacterial survival under antimicrobial treatments in farming practices, contributing to the emergence and pathogenicity of the ST34 *S.* Typhimurium monophasic variant.

## Supplementary Information

**Additional file 1. Bacterial strains and plasmids used in this study.**

**Additional file 2. Primers used in this study.**

**Additional file 3. The swimming motility of *Salmonella* strains**. Diameters of cell spread were measured 10 h post-inoculation.

**Additional file 4. Bacterial adhesion and invasion to IPEC-J2 cells**. The adhesion (A) and invasion (C) of the ST34 *Salmonella* and its mutant strains to IPEC-J2 cells. The adhesion (B) and invasion (D) of the ST19 *Salmonella* and its mutant strains to IPEC-J2 cells.

**Additional file 5. The histopathological analysis of liver from mice infected with various** ***Salmonella* strains**, including YZU0463 (B), YZU2855 (C), YZU0463Δ*fliC* (D), YZU2855Δ*fliC* (E), YZU2855^*fliC*→*fljB*^ (F). The results were compared with those from the control group (A), which did not undergo bacterial infection.
