# Supplementary material for: The predominant role of FliC contributes to the flagella-related pathogenicity of ST34 S. Typhimurium monophasic variant
Source: Vet Res. 2024 Dec 18;55:166. doi: 10.1186/s13567-024-01427-2 (PMC11654181; doi:10.1186/s13567-024-01427-2)
Supplement: Supplementary file 1 — Additional file 1. Bacterial strains and plasmids used in this study. [file 13567_2024_1427_MOESM1_ESM.docx]

# Additional file 1 Bacterial strains and plasmids used in this study

| **Strain or plasmid** | **Relevant characteristics** | **Reference** |
| --- | --- | --- |
| ***Escherichia coli*** | | |
| X7213 λ*pir* | Host for π requiring plasmids, conjugal donor | Laboratory collection |
| ***Salmonella* Typhimurium** | | |
| YZU0463 | ST34, expressing FliC or FljB | [18] |
| YZU0463Δ*fliC* | ST34, expressing FljB | This study |
| SL1344 | ST19, expressing FliC or FljB | Laboratory collection |
| SL1344Δ*fliC* | ST19, expressing FljB | This study |
| ***Salmonella* 4,[5],12:i:-** | | |
| YZU2855 | ST34, expressing FliC, derived from YZU0463 with *fljB* deleted | [18] |
| YZU2855Δ*fliC* | ST34, lacking flagellin | This study |
| YZU2855*^fliC^*^→^*^fljB^* | ST34, expressing FljB | This study |
| SL1344Δ*fljB* | ST19, expressing FliC | This study |
| SL1344Δ*fljB*Δ*fliC* | ST19, lacking flagellin | This study |
| SL1344Δ*fljB^fliC^*^→^*^fljB^* | ST19, expressing FljB | This study |
| **Plasmid** | | |
| pDM4 | Suicide vector, *pir* dependent, R6K, *SacBR*, Cm^r^ | Laboratory collection |
