# Supplementary material for: The predominant role of FliC contributes to the flagella-related pathogenicity of ST34 S. Typhimurium monophasic variant
Source: Vet Res. 2024 Dec 18;55:166. doi: 10.1186/s13567-024-01427-2 (PMC11654181; doi:10.1186/s13567-024-01427-2)
Supplement: Supplementary file 2 — Additional file 2. Primers used in this study. [file 13567_2024_1427_MOESM2_ESM.docx]

**Additional file 2: Primers used in this study**

| **Primer name** | **Primer sequence ( 5’ to 3’ )** | **Target** |
| --- | --- | --- |
| pDM4-F | GGTGCTCCAGTGGCTTCTGTTTCTA | For deletion mutants |
| pDM4*-R* | CAGCAACTTAAATAGCCTCTAAT | For deletion mutants |
| *fliC*-up-F | tgtggaatcccgggagagctcGTGCCATAGGCGGTTAGCTTT | For *fliC* deletion mutant |
| *fliC*-up-R | aatcgccggaGATCTTTTCCTTATCAATTACAACTTGAT | For *fliC* deletion mutant |
| *fliC*-down-F | ggaaaagatcTCCGGCGATTGATTCACCG | For *fliC* deletion mutant |
| *fliC*-down-R | aagcttatcgataccgtcgacTAGGTATGATGCGCCCGC | For *fliC* deletion mutant |
| *fliC*-out-F | tcgcccaactgctctttggt | For *fliC* deletion mutant |
| *fliC*-out-R | cgtactgctgttggcatggt | For *fliC* deletion mutant |
| *fliC*-in-F | cgcgaacatcaaaggtctga | For *fliC* deletion mutant |
| *fliC*-in-R | atgctgctttggcacaggtt | For *fliC* deletion mutant |
| *fljB*-up-F | aagcttatcgataccgtcgacCTGCGGCCCAGTCTGTCTAA | For *fljB* deletion mutant |
| *fljB*-up-R | AAAATTTTCCTTTTGGAAGGTTTTT | For *fljB* deletion mutant |
| *fljB*-down-F | ccttccaaaaggaaaattttTTATTTCGTTTTATTCAGCCCCG | For *fljB* deletion mutant |
| *fljB*-down-R | tgtggaatcccgggagagctcTTTAACTTCAATGCGCTTATTTCC | For *fljB* deletion mutant |
| *fljB*-out-F | actggtatcaatactatcgg | For *fljB* deletion mutant |
| *fljB*-out-R | accagcgaaagattaaaagc | For *fljB* deletion mutant |
| *fljB*-in-F | gagttaaaagatacaccggc | For *fljB* deletion mutant |
| *fljB*-in-R | gtggttttagcggctgcttc | For *fljB* deletion mutant |
| *fljB*-F | ggaaaagatcatggcacaagtaatcaacac | For *fliC* replacement mutant |
| *fljB*-R | tcgccggaTTAACGTAACAGAGACAGCACGTTC | For *fliC* replacement mutant |
| *fliC*-up-R2 | cttgtgccatGATCTTTTCCTTATCAATTACAACTTGAT | For *fliC* replacement mutant |
| *fliC*-down-F2 | ctgttacgttaaTCCGGCGATTGATTCACCG | For *fliC* replacement mutant |
| RT-*fljB*-F | GCTACGGGTGGTACGAATGG | For *fljB* expression analyse |
| RT-*fljB*-R | GCATCAGCACCAGTAAAGCC | For *fljB* expression analyse |
| RT-*fliC*-F | GGGGGAACTGGTAAAGATGGC | For *fliC* expression analyse |
| RT-*fliC*-R | GTAGTCCACCTGTAAGCGGG | For *fliC* expression analyse |
| RT-*gyrA*-F | AGATGTTGTCGTGACGCTGT | Reference gene for qPCR |
| RT-*gyrA*-R | TGGTGTCATGGGTGTTAGCC | Reference gene for qPCR |
