# Supplementary material for: The predominant role of FliC contributes to the flagella-related pathogenicity of ST34 S. Typhimurium monophasic variant
Source: Vet Res. 2024 Dec 18;55:166. doi: 10.1186/s13567-024-01427-2 (PMC11654181; doi:10.1186/s13567-024-01427-2)
Supplement: Supplementary file 3 — Additional file 3. The swimming motility of Salmonella strains. Diameters of cell spread were measured 10 h post-inoculation. [file 13567_2024_1427_MOESM3_ESM.pdf]

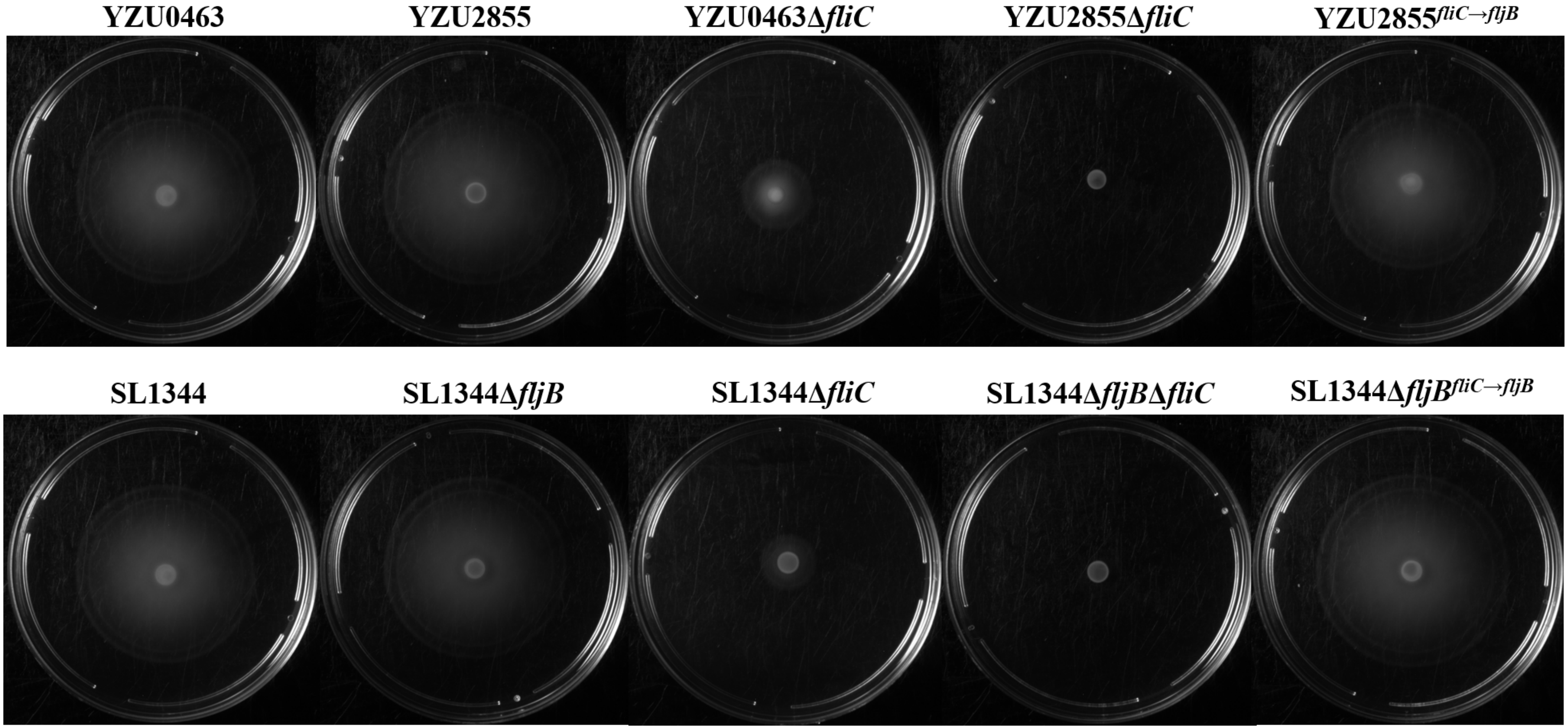
YZU0463
YZU2855
YZU0463Δ*fliC*
YZU2855Δ*fliC*
YZU2855$^{fliC\rightarrow fljB}$
SL1344
SL1344Δ*fljB*
SL1344Δ*fliC*
SL1344Δ*fljB*Δ*fliC*
SL1344Δ*fljB*$^{fliC\rightarrow fljB}$
