# Supplementary figures and images for: The predominant role of FliC contributes to the flagella-related pathogenicity of ST34 S. Typhimurium monophasic variant
Source: Vet Res. 2024 Dec 18;55:166. doi: 10.1186/s13567-024-01427-2 (PMC11654181; doi:10.1186/s13567-024-01427-2)

A

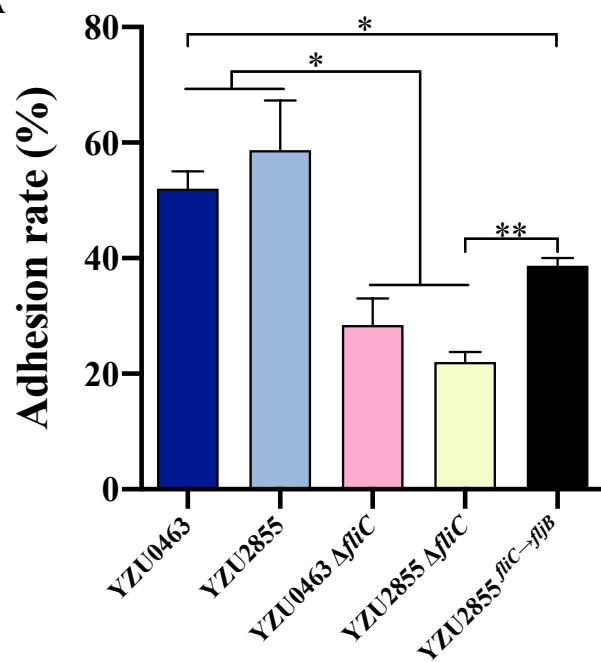

C

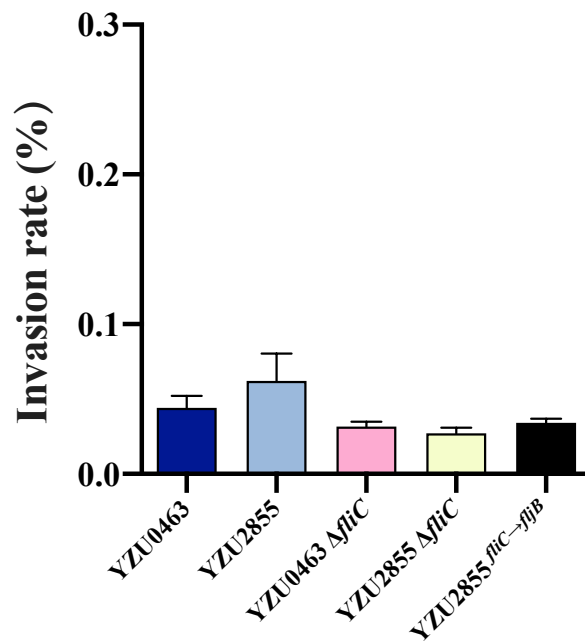

B

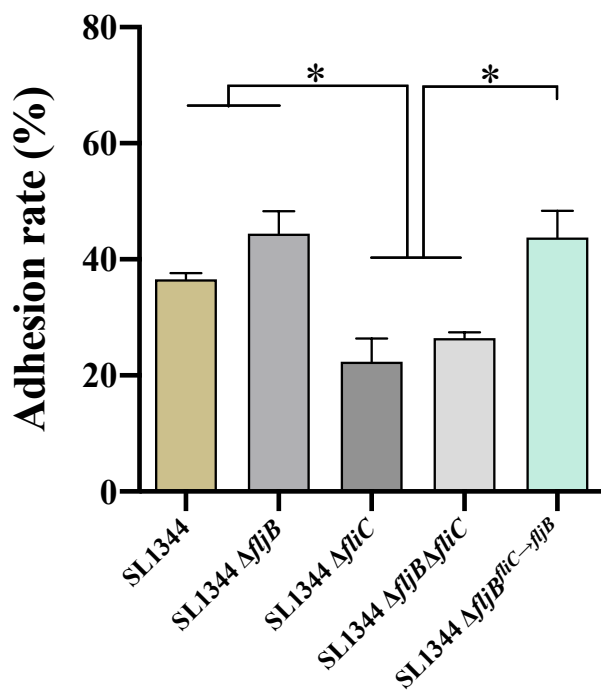

D

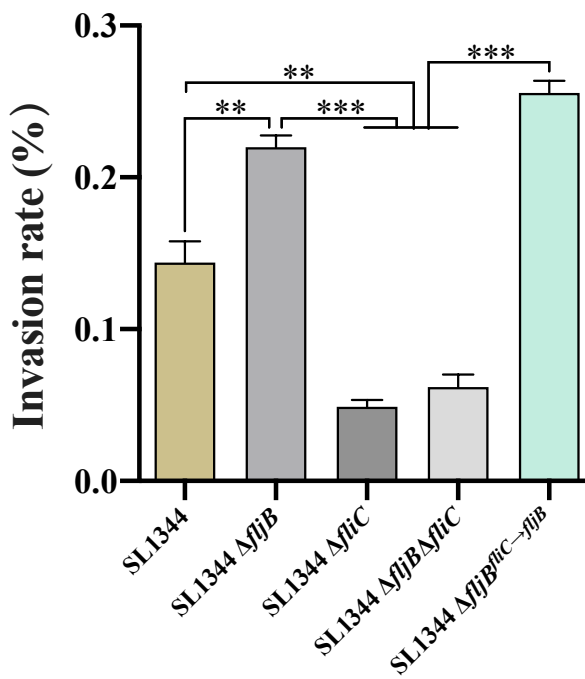

Supplement: Supplementary file 4 — Additional file 4. Bacterial adhesion and invasion to IPEC-J2 cells. The adhesion (A) and invasion (C) of the ST34 Salmonella and its mutant strains to IPEC-J2 cells. The adhesion (B) and invasion (D) of the ST19 Salmonella and its mutant strains to IPEC-J2 cells. [file 13567_2024_1427_MOESM4_ESM.pdf]

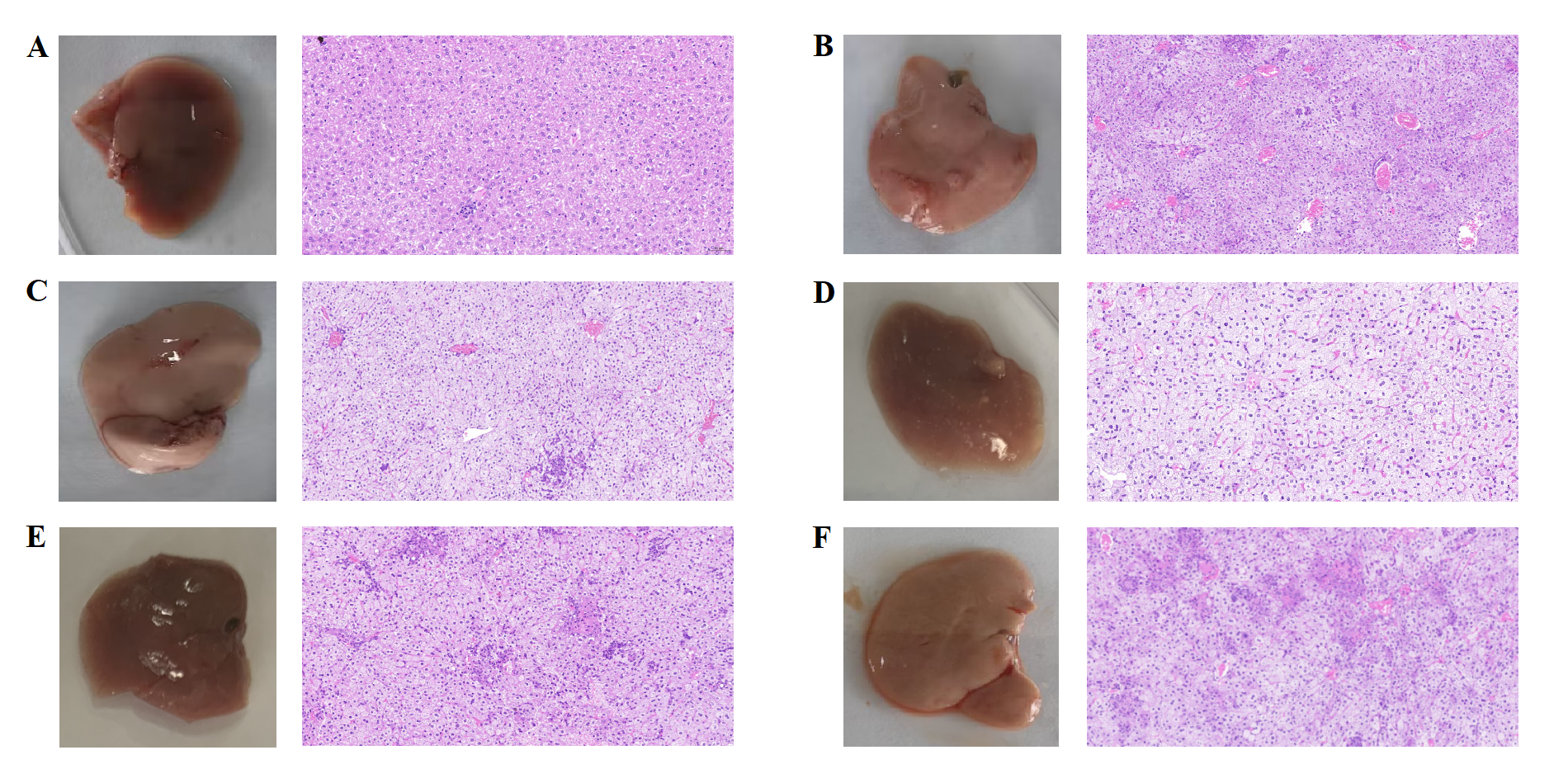

Supplement: Supplementary file 5 — Additional file 5. The histopathological analysis of liver from mice infected with various Salmonella strains, including YZU0463 (B), YZU2855 (C), YZU0463ΔfliC (D), YZU2855ΔfliC (E), YZU2855fliC→fljB (F). The results were compared with those from the control group (A), which did not undergo bacterial infection. [file 13567_2024_1427_MOESM5_ESM.tif]
